# Prevalence and Psychosocial Correlates of Depressive Symptoms in Urban Chinese Women during Midlife

**DOI:** 10.1371/journal.pone.0110877

**Published:** 2014-11-14

**Authors:** Carmen K. M. Wong, Jun Liang, Man L. Chan, Yin H. Chan, Laam Chan, Kwong Y. Wan, Ming S. Ng, Dicken C. C. Chan, Samuel Y. S. Wong, Martin C. S. Wong

**Affiliations:** 1 Division of Family Medicine and Primary Health Care, JC School of Public Health and Primary Care, The Chinese University of Hong Kong, 4/F, School of Public Health building, Prince of Wales Hospital, Shatin, New Territories, Hong Kong SAR; 2 The Hong Kong Primary Care Research Network, F2029A, Special Block, Tuen Mun Hospital, New Territories, Hong Kong SAR; Institute of Psychiatry, United Kingdom

## Abstract

**Objective:**

Depression is common in women with much research focusing on hormonal changes and menopausal symptoms but with little exploration of psychosocial problems in midlife. This study investigates the prevalence of clinically relevant depressive symptoms in midlife Chinese women and its association with psychosocial factors.

**Methods:**

A cross-sectional, community-based household survey of women aged 45 to 64 years of age was conducted in Hong Kong from September 2010 to March 2011. The structured questionnaire included demographic data, educational status, marital status and household income, as well as perceived current stressful events and significant life events in the past 12 months. Information on clinically relevant depressive symptoms was measured by the validated chinese Patient Health Questionnaire (PHQ-9).

**Results:**

A total of 402 participants were recruited in the study period. Of the 393 women who completed the questionnaire, the prevalence of clinically relevant depressive symptoms (PHQ-9 score≧10) was 11.0%. In multiple regression analysis, being single/divorced/separated/widowed, having an educational level of primary school level or below, having multiple chronic diseases, loss of hobby or loss of close social support in the past 12 months in midlife were associated with clinically relevant depressive symptoms.

**Conclusions:**

Correlates of clinically relevant depressive symptoms in midlife Chinese women can be used to identify those at increased risk and potentiate further studies to explore early psychosocial and community interventions.

## Introduction

### Women and depression

According to the World Health Organization [1], by the year 2020 depression will be the second most significant condition globally in terms of disability-adjusted life years lost. The lifetime prevalence of major depression ranges from 6–17% [2] and studies have consistently shown dysthymia, minor depression, brief recurrent depression and major depression to be more common in women compared to men. The prevalence of these conditions in women can range from 1.5 to 3 times to that of men [3], [4] and such differences may be consistent across life stages as women show higher rates of depression from the age range 11–15 to midlife [5]. Given that there are no gender differences in the course of depression e.g. illness duration, recovery time and recurrence risk [5], [6], social environmental factors are likely to have contributed to the early onset and increase in prevalence of depression in women.

### Midlife women

The risk of developing depression during the menopausal transition is high and associated with the presence of vasomotor symptoms, greater hormonal variability and changes in health and lifestyle behaviours [7]–[9]. A large, epidemiologically based study conducted in the US noted differences in psychological and vasomotor symptoms between caucasian and chinese midlife women. Symptoms of muscle and joint pain, and psychological symptoms such as lack of concentration, irritability, insomnia were more prevalent than vasomotor symptoms in Chinese women [9]. These findings were consistent with local and regional studies [10]–[12]. Although social and cultural factors are likely to play an important role in differences seen in psychological and depressive symptoms in midlife women, there has been little exploration of psychosocial correlates of depressive symptoms in women during their midlife years. In this study, a stratified household interview approach was selected to investigate the prevalence and psychosocial correlates of clinically relevant depressive symptoms in midlife women living within urban communities of Hong Kong.

## Material and Methods

### Study population and sampling frame

This study was a cross- sectional, population-based household survey of middle aged Chinese women (45 to 64 years old) in Hong Kong. Midlife women of age 45–65 constitute 30% of women in Hong Kong and 16% of the total Hong Kong Chinese population of over 7 million people [13]. Household surveys by cluster sampling has been the most frequently used sampling method as private and public housing blocks make up 99% of the residences of the population [14].

Geographically, Hong Kong is made up of Hong Kong Island, Kowloon and New Territories district, in which 18.0%, 29.8% and 52.2% of the population reside respectively. In this study, the community sample was drawn from the New Territories West district which comprises 1.88 million of the population and comparable to the Hong Kong population in terms of proportion of population of Chinese ethnicity (95.1% vs. 93.6%), post secondary educational attainment (23.9% vs. 25.9%), labour force participation (60.7% vs. 59.7%), median age (40.4 vs. 41.7), proportion never married (31.8% vs. 31.6%), average domestic household (3.0 vs. 2.9), median monthly household income ($20,040 vs. $20,500) and owner occupancy of residence (53.6% vs. 52.1%) [13].

A list of housing blocks in the Kwai Tsing, Tseun Wan, Tuen Mun and Yuen Long district which comprises the New Territories West district was drawn and stratified into private and public housing to avoid over sampling of public housing blocks. A total of three public housing estates and two private housing estates were included. Trained interviewers visited all households identified by computer generated random numbers in the cluster sample. Informed consent was obtained from eligible subjects (women aged 45–64) who agreed to participate in the study. Only one woman from each household was selected using the nearest date of birthday to interview date method. Eligible subjects who were unavailable for the study were visited on two further occasions. Interviews were conducted from the study period September 2010 to March 2011. The overall response rate was 60.0% and 402 participants were successfully interviewed.

### Ethics approval

Ethical approval was obtained from the Joint NTWC Clinical Research Ethical Committee (Ref. no.: NTWC/CREC/883/10) before the start of the trial. Written formal consent was obtained and participants were able to withdraw from the survey at any time.

### Questionnaire

A structured questionnaire was administered face-to-face by trained interviewers to each participant. The questionnaire included information on socio-demographic data such as age, gender, marital status, educational attainment, employment status, median monthly household income as well as lifestyle history (smoking, alcohol) and medical history (general health, chronic disease and significant life events). The Patient Health Questionnaire (PHQ-9) survey was used to assess clinically relevant depressive symptoms. The PHQ-9 is a nine item depression scale of the PHQ. A score of 5 and above indicate depressive symptoms which are mild and warrant watchful waiting, a score of 10 to 14 indicates clinically relevant moderate depression, which warrant initiation of counselling and/or medication and a score of 15 and above indicates moderately severe symptoms requiring medication. The PHQ-9 has widespread clinical use in primary care clinics in the screening and monitoring of depression and it has been validated in Chinese [15]. The cutoff score of 10 and above has a sensitivity of 88% and a specificity of 88% for major depression [16].

In addition, the survey encompass the 8-question modified Life Event scale to capture important life events that occurred in the previous 12 months [17]. These events included: serious illness or accidents in spouse, death of a close relative or friend, separation from a child, close friend or relative to whom the subject depend on for help, loss of a pet, giving up of an important hobby, experience of serious financial difficulties, move of residence and any other significant events, either good or bad.

### Statistical Methods and Analysis

Sample size calculation was based on the assumption of achieving a 5% precision level and a conservative assumption of depression prevalence of 50% to yield the greatest number of subjects. Using 4×p(1- p)/(precision)^2^ where p  =  estimated proportion of participants having depressive symptoms, the minimal sample needed was 383 participants.

All data were entered and analyzed in PASW Statistics version 18.0. The primary outcome was the prevalence of depressive symptoms defined as PHQ-9 score ≥10. Each of the socio-demographic, lifestyle and clinical variables listed above was consecutively tested against the outcome variable by bivariate analysis. All p-values ≤0.05 were regarded as statistically significant in the comparative analysis.

## Results

A total of 402 participants were recruited in the study period, of which 393 women completed the questionnaire. Table 1 illustrates the demographic and lifestyle characteristics of the study population.

**Table 1:** Socio-demographic Characteristics.

		N = 402 (%)
**Age groups**		
	45–50 years	164 (40.8%)
	51–54 years	96 (23.9%)
	55–60 years	104 (25.9%)
	61–64 years	38 (9.5%)
**Mean age in years (sd)**		52.5 (5.3)
**Mean duration of residence in Hong Kong (sd)**		29.0 (19.2)
**Residence**		
	Living alone	15 (3.9%)
	Living with spouse/ parents/ children/ siblings	368 (96.1%)
**Marital Status**		
	Single/Divorced/Separate/Widowed	72 (18.9%)
	Married/cohabit	308 (81.1%)
**Mean number of children (sd)**		2.3 (1.1)
**Educational level**		
	Primary school and below	170 (44.5%)
	Secondary school and above	212 (55.5%)
**Job**		
	Full time	53 (23.5%)
	Part time	62 (27.4%)
	Housewife	111 (49.1%)
**Monthly Household income (HK$)**		
	<10,000	173 (56.2%)
	10,000–19,999	32 (10.4%)
	≥ 20,000	9 (2.9%)
	Not known/ refused to respond	94 (30.5%)
**Menopausal Status**		
	Pre/ Perimenopausal	236 (58.7%)
	Postmenopausal	166 (41.3%)
**Number of chronic diseases**		
	0	213 (60.9%)
	1	79 (22.6%)
	2+	58 (16.6%)
**Medical History**		
	Cardiovascular disease (MI/Stroke)	11 (2.7%)
	Hypertension	53 (13.2%)
	Diabetes	28 (7.0%)
	Depression/Anxiety	24 (6.0%)
**General health status**		
	Excellent	4 (1.0%)
	Very good	57 (14.4%)
	Good	93 (23.5%)
	Fair	207 (52.4%)
	Poor	34 (8.6%)
**Patient Health Questionnaire (PHQ-9)**		
	0–4 points	255(64.9%)
	5–9 points	95 (24.2%)
	10–14 points	25 (6.4%)
	15+ points	18 (4.6%)

The mean age of the participating women was 52.5 years (s.d. 5.3) and had been resident in Hong Kong for 29.0 years. Of the 402 women, the majority (81%) were married or in cohabiting relationships, and lived with family members (96%). Educational levels at secondary school level or above was obtained in over half of the women (56%). Approximately half of those interviewed (49%) were housewives without salaried jobs. Overall, women had on average 2 children (mean 2.3, s.d. 1.1). Up to 56% had median household income lower than HK$10,000. The majority of women were non smokers (95%) and non alcohol drinkers (94%). Over half of women rated their general health as fair or poor (52% and 9% respectively) whilst the rest rated their health from good to excellent. Over 60% of women were free from chronic illnesses, whilst 22% suffered from one chronic condition and 17% suffered from two or more conditions. Only 6.0% (n = 24) had a self reported diagnosis of depression or anxiety.

### Prevalence of clinically significant depressive symptoms and psychosocial and lifestyle correlates

Using PHQ-9 as the measuring instrument with a cut-off level of 10 points and above, 11.0% of participants (n = 43) suffered from clinically relevant depressive symptoms indicative of major depression. Of those, 18 participants scored 15 points and above indicating severe depressive symptoms.

For socioeconomic factors, being single/divorced/separated/widowed, having an educational level of primary school level or below, having poorer reported general health and having multiple chronic diseases were significantly associated with clinically relevant depressive symptoms. There were no significant associations of depressive symptoms with early to late midlife age groups, living alone, number of children, employment status, monthly household incomes, menopausal status, smoking or drinking. Psychosocial correlates of depressive symptoms included having experienced two or more stressful life events in the past 12 months, of which “serious illness or accident of spouse or partner”, “separation from close friend or relative”, “loss of a hobby”, “serious financial trouble” and “experience in either a good or bad important event” was significant. Current worries regarding marriage, children, finance, job or health had no correlation with depressive symptoms (see table 2).

**Table 2:** Bivariate associations of those with clinically depressive symptoms (PHQ 10+) and those without (PHQ <10) on socio- demographic characteristics and stressful life events.

		n	Prevalence of depression (n, %)	P- value
**Age group**				
	45–54 years	252	29 (11.5%)	0.631 (χ^2^ = 0.231, df = 1)
	55–64 years	141	14 (9.9%)	
**Marital Status**				
	Single/Divorced/Separate/Widowed	72	16 (22.2%)	<0.001 (χ^2^ = 12.653, df = 1)
	Married/cohabit	305	24 (7.9%)	
**Residence**				
	Living alone	15	3 (20.0%)	0.223^a^
	Living with spouse/parents/children/siblings	365	39 (10.7%)	
**Number of children**				
	None	12	1 (8.3%)	0.594 (χ^2^ = 1.041, df = 2)
	1	62	9 (14.5%)	
	2+	302	31 (10.3%)	
**Educational level**				
	Primary school and below	170	25 (14.7%)	0.043 (χ^2^ = 4.109, df = 1)
	Secondary school and above	209	17 (8.1%)	
**Job**				
	Full time	53	8 (15.1%)	0.323 (χ^2^ = 2.262, df = 2)
	Part time	61	4 (6.6%)	
	Housewife	111	14 (12.6%)	
**Monthly Household income (HK$)**				
	<10,000	173	24 (13.9%)	0.256 (χ^2^ = 1.292, df = 1)
	10,000 +	41	3 (7.3%)	
**Smoking status**				
	Non-smoker	349	36 (10.3%)	0.759 (χ^2^ = 0.094, df = 1)
	Current smoker/Past smoker	13	1 (7.7%)	
**Drinking status**				
	Non-drinker	332	33 (9.9%)	0.897 (χ^2^ = 0.017, df = 1)
	Current drinker	22	2 (9.1%)	
**General health status**				
	Excellent/Very good/Good	152	4 (2.6%)	<0.001 (χ^2^ = 17.164, df = 1)
	Fair/Poor	238	38 (16.0%)	
**Menopausal Status**				
	Pre/Perimenopausal	228	23 (10.1%)	0.524 (χ^2^ = 0.406, df = 1)
	Postmenopausal	165	20 (12.1%)	
**Number of chronic diseases**				
	0	210	11 (5.2%)	<0.001 (χ^2^ = 36.483, df = 2)
	1	79	7 (8.9%)	
	2+	58	19 (32.8%)	
**Number of stressful life events**				
	0–1	272	21 (7.7%)	0.001 (χ^2^ = 10.220, df = 1)
	2 or above	117	22 (18.8%)	
**Stressful life event**				
	Serious illness or accident of spouse or partner	31	9 (29.0%)	0.004^a^
	Death of close relative or friend	92	15 (16.3%)	0.075 (χ^2^ = 3.173, df = 1)
	Separated from close friend or relative whom depend on for help	31	9 (29.0%)	0.003^a^
	Loss of pet	20	1 (5.0%)	0.710^a^
	Loss of a hobby	70	17 (24.3%)	<0.001 (χ^2^ = 14.837, df = 1)
	Serious financial trouble	65	13 (20.0%)	0.010 (χ^2^ = 6.650, df = 1)
	Moving home	13	2 (15.4%)	0.645^a^
	Experience in either good or bad important events	103	18 (17.5%)	0.012 (χ^2^ = 6.363, df = 1)
**Most concerned issues**				
	Marriage	8	1 (12.5%)	0.887 (χ^2^ = 0.020, df = 1)
	Children	151	15 (9.9%)	0.613 (χ^2^ = 0.256, df = 1)
	Finance	82	13 (15.9%)	0.109 (χ^2^ = 2.566, df = 1)
	Job	30	6 (20.0%)	0.121^a^
	Health	176	20 (11.4%)	0.809 (χ^2^ = 0.058, df = 1)
	Others	27	0 (0%)	-

Chi-squared test was applied unless indicated otherwise.

Note: ^a^ Fisher's exact test.

### Multivariate model

All variables found to be significantly associated with clinically relevant depressive symptoms were entered into the multivariate logistic regression model. Factors such as not being in a married or cohabiting relationship, having primary school education of lower, having multiple co-morbidity and life events such as “loss of hobby” or “separated from close friend or relative whom depend on for help”, was independently associated with having clinically relevant depressive symptoms (see table 3).

**Table 3:** Multivariate regression model prevalence odds ratio for those with clinically depressive symptoms (PHQ 10+) versus those without (PHQ <10).

Variable		Odds ratio (95% CI)
**Marital Status**		
	Married/Cohabit	1
	Single/Divorced/Separate/Widowed	**2.45 (1.04–5.80)^a^**
**Educational level**		
	Secondary school and above	1
	Primary school and below	**2.39 (1.07–5.34)^a^**
**Number of chronic medical conditions**		
	0	1
	1	1.59 (0.56–4.53)
	2 or above	**7.66 (3.04–19.31)^a^**
**Stressful life event**		
	Serious illness or accident of spouse or partner	1.06 (0.33–3.48)
	Death of close relative or friend	1.29 (0.53–3.10)
	Separated from close friend or relative whom depend on for help	**3.69 (1.11–12.23)^a^**
	Loss of a hobby	**2.58 (1.06–6.25)^a^**
	Serious financial trouble	1.50 (0.56–3.98)
	Experience in either good or bad important events	0.81 (0.33–1.99)

Note: ^a^ p-value <0.05.

## Discussion

This was the first household survey conducted in midlife Chinese women to investigate the prevalence and correlates of depressive symptoms with psychosocial factors using a validated screening tool for depressive symptoms.

The prevalence of clinically relevant depressive symptoms in Chinese midlife women was 11.0%. Studies have suggested ethnic differences, in which Chinese midlife women had lower odds than white women to have significant depressive symptoms [18], although our study results showed higher prevalence in comparison to US adult women (8.55% score ≧10 using PHQ-9) [19]. However, comparison of prevalence data can be problematic owing to the differences in sampling framework, screening methods used, cutoff levels and in the few studies reporting data relating to women in midlife years.

Previous studies have shown that lower socioeconomic status was associated with higher rates of depression in women [20]–[24]. Although our study showed that depressive symptoms in midlife women were significantly associated with lower educational level, there was no association with lower household income or employment status. There may be several reasons for this perhaps 1) socioeconomic status play a minimal role in the occurrence of depressive symptoms in midlife or 2) socioeconomic inequalities may be buffered from household members income as 93% of women live with others and/or 3) socio-demographic parameters used in this study are not extensive or sensitive enough to detect individual social economic situations e.g. social relationships, financial strain, level of debt etc.

Previous studies have shown that stressful life events can precipitate the onset of depressive symptoms [25], [26]. A similar community-based cross-sectional household survey conducted in Hong Kong show that midlife urban Chinese men who were single/divorced/separated/widowed, living alone and unemployed increased their vulnerability for clinically relevant depressive symptoms [27]. Whilst negative events such as “given up a hobby or activity” and “serious financial trouble” were independently associated with depressive symptoms in men; in our study of midlife women, living alone and employment status had no effect on depressive symptoms but “loss of a hobby or activity” and “separation from a close friend or relative” was more likely in women to have clinically relevant depressive symptoms. The effect of gender traditional roles [28] and women's tendency to “tend and befriend” [29]may explain these study findings. Traditional expectations of men to be the breadwinner may explain the negative effect of unemployment, financial trouble in men, whilst women's interpersonal and nurturing nature may explain the importance of close social support for women as a coping strategy. This is also consistent with the ‘cost of caring’ theory by Kessler and McRae [30] and Turner and Avison [31] who hypothesized that women tended to care more for others and thus were more vulnerable to events affecting close emotional ties rather than themselves. One study of couples with shared life events had suggested life events relating to children, housing or reproductive problems were specific in the onset of depression in women [32]. In our study of midlife women, these were not apparent and current worries regarding marriage, children, finance, job or health had no correlation with depressive symptoms. In midlife, changes such as the loss of hobby or activity in the past year and having poorer general health may highlight other physical or psychosocial pressures specifically facing those in midlife.

Hong Kong has a high life expectancy of 85.9 years, second to that of Japan [33] with midlife and elderly women making a substantial and increasing proportion of the population. The prevalence of depressive symptoms in the Chinese population appear to increase with age [34] and in the Chinese elderly age 70 years and above, significant depressive symptoms rise in both men and women [35], [36], consistent with epidemiological studies which depict lower rates of depressive symptoms in midlife and elevated rates in the oldest old [37]. Differences in the prevalence of depressive symptoms in midlife and in older years may be attributable to the use of different assessment scales, patterns of health care utilization, acquisition of coping skills, cohort effect of those born at an earlier age, or period effect of different social environmental influences. Thus, there is a need for longitudinal cohort studies which can help us understand and identify those at risk of depression from the transition from midlife to elder years.

The association of poor physical health and comorbidity with depression is well documented [38], [39], particularly in the older age community [35], [36]. This association is also apparent in our study of midlife women, not surprisingly so, as this period is a vulnerable time for the onset of many chronic physical conditions.

Women that are married or in co-habiting partnerships in midlife appear to have less significant depressive symptoms. As midlife women play increasingly complex roles in their families and careers in urban societies, further studies exploring depressive symptoms and depression in midlife men and women are needed. The socio-demographics of Chinese midlife women in Hong Kong are changing with increasing numbers of women who have never married or are divorced or without children [13]. This is likely to impact the dynamic pattern of depressive symptoms in midlife women and close monitoring is required so that psychosocial interventions can be responsive and effective.

### Limitations

This study was conducted in urban communities of Hong Kong. The demographic data of women in our sample compared to age-adjusted data of the census of the whole territory showed higher rates of low educational attainment to primary school level (44.5% vs. 34.1%) although comparative rates of employment (50.9% vs. 48.8%). Selection bias inherent in our study in selecting those of lower educational attainment may result in the overestimation of depressive symptoms and may not be representative of the whole region. In addition, the questionnaire was only conducted at one time point. It is unclear whether depressive symptoms are of new onset, persisting, recurrent or likely to resolve. The PHQ-9 was the screening tool used for detecting depressive symptoms in this study, and there are many other tools for this purpose which may yield a slight variation in prevalence results. The PHQ-9 and its shorter version PHQ-2, however is widely used clinically by health care professionals to screen for depression as a self administered tool or face to face interview and our study population would likely reflect the population that have detectable clinically relevant depressive symptoms in primary care.

### Conclusion and Recommendation

The prevalence of clinically depressive symptoms in this study of urban midlife Chinese women was 11.0%. In midlife, being single/divorced/separated/widowed, having an educational level of primary school level or below, having multiple chronic diseases, loss of hobby or loss of close social support in the past 12 months were associated with clinically relevant depressive symptoms. Further studies of depressive symptoms in midlife are necessary to identify those at risk and to explore early psychosocial and community interventions to improve mental health for midlife women.
